# Tumor microenvironment and breast cancer survival: combined effects of breast fat, M2 macrophages and hyaluronan create a dismal prognosis

**DOI:** 10.1007/s10549-019-05491-7

**Published:** 2019-11-12

**Authors:** Satu Tiainen, Amro Masarwah, Sanna Oikari, Kirsi Rilla, Kirsi Hämäläinen, Mazen Sudah, Anna Sutela, Ritva Vanninen, Juho Ikonen, Raija Tammi, Markku Tammi, Päivi Auvinen

**Affiliations:** 1 Cancer Center, Kuopio University Hospital, P.O. Box 100, 70029 Kuopio, Finland; 2 Institute of Clinical Medicine, University of Eastern Finland, P.O. Box 1627, 70211 Kuopio, Finland; 3 Imaging Center, Clinical Radiology, Kuopio University Hospital, P.O. Box 100, 70029 Kuopio, Finland; 4 Institute of Biomedicine, University of Eastern Finland, P.O.Box 1627, 70211 Kuopio, Finland; 5 Imaging Center, Clinical Pathology, Kuopio University Hospital, P.O. Box 100, 70029 Kuopio, Finland; 6 Institute of Clinical Medicine, Clinical Pathology and Forensic Medicine, University of Eastern Finland, P.O. Box 1627, 70211 Kuopio, Finland; 7 Biocenter Kuopio and Cancer Center of Eastern Finland, University of Eastern Finland, P.O. Box 1627, 70211 Kuopio, Finland; 8 Institute of Clinical Medicine, Clinical Radiology, University of Eastern Finland, P.O. Box 1627, 70211 Kuopio, Finland

**Keywords:** Breast cancer, Macrophage, Breast density, Hyaluronan, Diabetes, Obesity

## Abstract

**Purpose:**

Tumor microenvironment, including inflammatory cells, adipocytes and extracellular matrix constituents such as hyaluronan (HA), impacts on cancer progression. Systemic metabolism also influences tumor growth e.g. obesity and type 2 diabetes (T2D) are risk factors for breast cancer. Here, in 262 breast cancer cases, we explored the combined impacts on survival of M2-like tumor associated macrophages (TAMs), the abundance of breast fat visualized as low density in mammograms, and tumor HA, and their associations with T2D.

**Methods:**

Mammographic densities were assessed visually from the diagnostic images and dichotomized into very low density (VLD, density ≤ 10%, “fatty breast”) and mixed density (MID, density > 10%). The amounts of TAMs (CD163+ and CD68+) and tumor HA were determined by immunohistochemistry. The data of T2D was collected from the patient records. Statistical differences between the parameters were calculated with Chi square or Mann–Whitney test and survival analyses with Cox’s model.

**Results:**

A combination of fatty breasts (VLD), abundance of M2-like TAMs (CD163+) and tumor HA associated with poor survival, as survival was 88–89% in the absence of these factors but only 40–47% when all three factors were present (*p* < 0.001). Also, an association between T2D and fatty breasts was found (*p* < 0.01). Furthermore, tumors in fatty breasts contained more frequently high levels of M2-like TAMs than tumors in MID breasts (*p* = 0.01).

**Conclusions:**

Our results demonstrate a dramatic effect of the tumor microenvironment on breast cancer progression. We hypothesize that T2D as well as obesity increase the fat content of the breasts, subsequently enhancing local pro-tumoral inflammation.

**Electronic supplementary material:**

The online version of this article (10.1007/s10549-019-05491-7) contains supplementary material, which is available to authorized users.

## Introduction

Obesity is a major global problem and often a consequence of the Western lifestyle with high-energy diet and low level of physical activity. Obese individuals commonly develop insulin resistance, a condition that precedes type 2 diabetes (T2D). Obesity and T2D are both risk factors for breast cancer and they also predispose breast cancer patients to a poor outcome. [1–6].

In obese individuals, adipocytes in breast tissue induce the recruitment of inflammatory cells, especially macrophages [7], which in turn maintain a low-level inflammation [8]. Crown-like structures (CLSs) i.e. macrophages located around dead adipocytes are regarded as biomarkers of this white adipose tissue inflammation [9]. Among breast cancer patients, a high level of CLSs in breast tissue associates with a poor outcome [10]. Indeed, chronic low-level inflammation is one of the hallmarks of cancer [11], and several studies have shown, that high numbers of tumor associated macrophages (TAMs) correlate with tumor aggressiveness and a poor outcome in breast cancer [12, 13]. In addition, a positive correlation has been detected between body mass index (BMI) and the amount of TAMs in the breast cancer microenvironment [13].

Hyaluronan (HA) is a large extracellular glycosaminoglycan and a very interesting molecule in terms of obesity, T2D and inflammation since its synthesis depends on the availability of UDP-sugars, which increase in conjunction with high glucose uptake [14], and the expression of hyaluronan synthases HAS1-3 [15] upregulated by the cytokines and growth factors released during inflammation. Consequently, HA is abundant at sites of inflammation, and mainly via its receptors CD44 and RHAMM, HA can modulate inflammatory responses [16, 17] including the recruitment of macrophages in both adipose tissue [18] and breast cancer [13, 19]. Moreover, it seems that HA can induce macrophage polarization into the pro-tumoral M2-like phenotype [20, 21]. HA facilitates breast cancer progression [22] and interestingly, the abundance of tumor HA and obesity exhibit a mutual correlation in breast cancer patients [23].

Mammographic breast density (MBD) describes the composition of breast tissue i.e. the relative proportions of fibroglandular and adipose tissues. A high MBD is a well-established risk factor for breast cancer [24, 25] but the impact of MBD on breast cancer survival is less clear since the findings in different studies have been conflicting [25]. It was previously suggested that breast cancer patients with very low density breasts (VLD, density ≤ 10%), i.e. breasts abundant with fat, had a poor outcome, and VLD represented a significant negative prognostic factor even after correcting for potential confounding factors including age, menopausal status and BMI [26]. Interestingly, VLD correlated also with a high tumor HA content [27].

In the present material of 262 breast cancer patients, we explored the combined impacts on survival of fatty breasts (VLD), M2-like (CD163+) TAMs and tumor HA, and their possible associations with T2D. We hypothesized that fatty breasts, numerous M2-like TAMs and HA abundance together would create inflammatory conditions that promote tumor progression, resulting in poor outcome. In addition, we hypothesized that the disturbed energy metabolism encountered in T2D and obesity would favor this pro-tumoral inflammation in the tumor microenvironment.

## Materials and methods

### Patient material

The primary material of this retrospective study consisted of 278 breast cancer cases, of which 262 patients with data available of both MBD and the amount of TAMs were included in this study. The patients had been operated due to breast cancer at Kuopio University Hospital during the years 2002–2008. Half of the cases were HER2 positive and half were HER2 negative with matching age and time of surgery [23]. The follow-up data were updated in September 21, 2016. The Ethics Committee of the University of Eastern Finland (February 24, 2009, 19//2009), and the National Supervisory Authority for Welfare and Health (VALVIRA, April 8, 2009, 1928/05.01.00.06/2009) provided ethical approval for this study. The study was conducted in accordance with the Declaration of Helsinki.

### Type 2 diabetes and obesity

The data of types 1 and 2 diabetes at the time of breast cancer diagnosis were collected retrospectively from the patient records. Height and weight of the patients were measured for the primary breast cancer operation and collected from anesthesia forms, providing reliable preoperative values [23]. BMI was calculated by the following formula: bodyweight (kg) divided by square of the height (m). According to the WHO classification obesity was determined as BMI ≥ 30 kg/m^2^.

### Mammographic breast density

Mammograms were available for 270 patients and MBD was evaluated from the cranio-caudal projections of the original diagnostic mammograms, as reported previously [26]. Briefly, the percentage of the area occupied by radiologically dense breast tissue in the mammogram was evaluated visually. For this study, breast densities of ≤ 10% were classified as VLD (“fatty breast”) and > 10% as mixed densities (MID).

### Tumor associated macrophages

CD163 positivity was regarded as an indicator for M2-like TAMs, and CD68 positivity as an indicator for all TAMs. Immunohistochemical staining for TAMs and their evaluation were performed as described previously [13] with 276 and 270 adequate CD163 and CD68 immunostained tissue sections, respectively, available for analysis. Briefly, three investigators counted the TAMs in at least four hot spots and the average value represented the number of TAMs in the section. The levels of CD163+ and CD68+ TAMs were classified as either low or high; values lower or equal than the median were graded as “low”, and values higher than the median as “high”.

### Hyaluronan

The stainings of HA in breast carcinoma cells and adjacent stroma were performed as reported earlier [23]. In the statistical analyses, HA in breast carcinoma cells was classified as weak or strong (≤ 50% and > 50% stained cells, respectively), and stromal HA as weak or strong according to the intensity of the staining.

### The standard histopathological factors

Tumor size, nodal status, histopathological grade and type of the tumor (i.e. ductal, lobular etc.), estrogen (ER) and progesterone (PR) receptor status (immunohistochemistry) and HER2 expression (chromogenic in situ hybridization test) were determined in Kuopio University Hospital, Department of Pathology at the time of diagnosis in accordance with the WHO and international guidelines [28].

### Statistical analyses

The statistical analyses were performed with IBM SPSS Statistics 22 for Windows (IBM Corporation, Armonk, NY, USA). Chi square test and Mann–Whitney test were utilized to calculate the differences between the parameters. Univariate survival analyses were calculated with Cox’s model, and survival curves were plotted with the Kaplan–Meier method. Cox’s model was used also for multivariate survival analyses; the variables included were MBD, CD163+ and CD68+ TAMs, HA in breast carcinoma cells, stromal HA, BMI, T2D, tumor size (T2-4 vs. T1), nodal status (N1–3 vs. N0), ER and HER2 status. Overall survival (OS) and breast cancer specific survival (BCSS) were calculated from the date of diagnosis to death or end of follow-up; death from any cause was included as an event for OS and death from breast cancer for BCSS. Disease free survival (DFS) was calculated from the date of diagnosis to disease recurrence, death or end of follow-up; only disease recurrence was included as an event. *p* values ≤ 0.05 were considered statistically significant.

## Results

### Characteristics of the cases

The clinicopathological parameters of the 262 cases with data available of all the assays are presented in Tables 1 and 2. The median follow-up time was 9.7 years (range 0.5–15.2 years). During the follow up, 78 patients (30%) had a relapse and 63 (24%) developed distant metastases. Overall, 70 patients (27%) had died; 52 of them due to breast cancer and 18 from other causes.

**Table 1:** The standard histopathological parameters

Tumor classification, *n* (%)	
pT1	145 (55%)
pT2	94 (36%)
pT3	10 (4%)
pT4	13 (5%)
Nodal classification, *n* (%)	
pN0	96 (37%)
pN1	116 (44%)
pN2	34 (13%)
pN3	16 (6%)
Histological grade, *n* (%)	
1	22 (8%)
2	113 (43%)
3	127 (49%)
Tumor histology, *n* (%)	
Ductal	214 (82%)
Lobular	26 (10%)
Mucinous	4 (1%)
Other	18 (7%)
HER2 status, *n* (%)	
Positive	129 (49%)
Negative	133 (51%)
ER status, *n* (%)	
Positive	188 (72%)
Negative	74 (28%)
PR status, *n* (%)	
Positive	163 (62%)
Negative	99 (38%)

*ER* estrogen receptor, *PR* progesterone receptor

**Table 2:** Characteristics of the cases

Age, years	
Median	58.7
Range	32–86
BMI (kg/m^2^), *n* (%)	
< 30	207 (79%)
≥ 30	55 (21%)
T2D, *n* (%)	
No	227 (87%)
Yes	35 (13%)
Relapse, *n* (%)	
No	184 (70%)
Yes	78 (30%)
Death, *n* (%)	
No	192 (73%)
Yes	70 (27%)
MBD, *n* (%)	
MID	166 (63%)
VLD	96 (37%)
CD163+ TAMs, *n* (%)	
Low	134 (51%)
High	128 (49%)
CD68 + TAMs, *n* (%)	
Low	136 (52%)
High	126 (48%)

*BMI* body mass index, *T2D* type 2 diabetes, *MBD* mammographic breast density; *MID* mixed density, *VLD* very low density, *TAMs* tumor associated macrophages

Among the 262 cases, 37% had VLD breasts and the remaining 63% had MID breasts (Table 2). High levels of M2-like (CD163+) TAMs were detected in 49% and high levels of CD68+ TAMs in 48% of the tumors (Table 2). T2D was present in 13% of the patients; none of the patients had type 1 diabetes (Table 2). Half (51%, *n* = 18) of the type 2 diabetics were also obese. Of the T2D patients, 66% (*n* = 23) were treated with metformin (19 only metformin and 4 metformin with insulin), 6% (*n* = 2) with other oral T2D medication, 3% (*n* = 1) with insulin alone, 23% (*n* = 8) were not receiving medication for T2D and for one patient the information of T2D treatments was missing. At the time of diagnosis, 21% of all the patients were obese (BMI ≥ 30) (Table 2). The standard pathological factors such as tumor size, nodal status, hormone receptor status and HER2 status were similar among the obese and non-obese patients (data not shown). In line with previous data [26], an association was found between obesity and VLD breasts (*p* < 0.01) (Table 3). Also, the median for the number of CD163+ TAMs was higher in tumors of obese compared to tumors of non-obese patients, 29 (range 10–58) and 25 (range 5–65), respectively (*p* = 0.032), but there was no correlation between CD68+ TAMs and BMI (*p* = 0.6).

**Table 3:** The correlations between breast density and TAMs, T2D and BMI

	VLD *n* = 96 *n* (%)	MID *n* = 166 *n* (%)	*p* value
CD163+ TAMs			
Low (*n* = 134)	39 (41%)	95 (57%)	
High (*n* = 128)	57 (59%)	71 (43%)	0.01
CD68 + TAMs			
Low (*n* = 136)	49 (51%)	87 (52%)	
High (*n* = 126)	47 (49%)	79 (48%)	0.831
T2D			
No (*n* = 227)	70 (73%)	157 (95%)	
Yes (*n* = 35)	26 (27%)	9 (5%)	< 0.01
BMI (kg/m^2^)			
< 30 (*n* = 207)	63 (66%)	144 (87%)	
≥ 30 (*n* = 55)	33 (34%)	22 (13%)	< 0.01

*TAMs* tumor associated macrophages, *BMI* body mass index, *T2D* type 2 diabetes, *VLD* very low density, *MID* mixed density

### High level of M2-like TAMs associates with VLD breasts

Tumors in VLD breasts contained more often high levels of M2-like TAMs than tumors in MID breasts, i.e. 59% and 43%, respectively (*p* = 0.01) (Table 3). Similarly, among the non-obese patients (*n* = 207), high levels of M2-like TAMs occurred in 57% and 41% of the tumors in VLD and in MID breasts, respectively (*p* = 0.032). Among the obese patients (*n* = 55), high levels of M2-like TAMs occurred frequently both in the tumors in VLD and in MID breasts, i.e. 64% and 55%, respectively (ns). No correlation was found between breast density and the amount of CD68+ TAMs (ns) (Table 3).

### Type 2 diabetes associates with VLD breasts

An association was found between T2D and VLD breasts, since 27% of the patients with VLD breasts but only 5% of the patients with MID breasts had T2D (*p* < 0.01) (Table 3). Among the non-obese patients, T2D was found in 19% and 4% of the patients with VLD and MID breasts, respectively (*p* < 0.01). Among the obese patients, T2D occurred in 42% and 18% of the patients with VLD and MID breasts, respectively (*p* = 0.061). No correlations were found between T2D and the amounts of TAMs or HA (data not shown). A non-significant trend towards lower tumor HA among T2D patients with metformin medication (*n* = 23) compared to those without metformin (*n* = 11) was found, but there were no correlations between TAMs and T2D treatments (Supplementary Table S1).

### Fatty breasts (VLD), a high level of M2-like TAMs and high tumor HA create a risk for dismal outcome

**Fig. 1:**
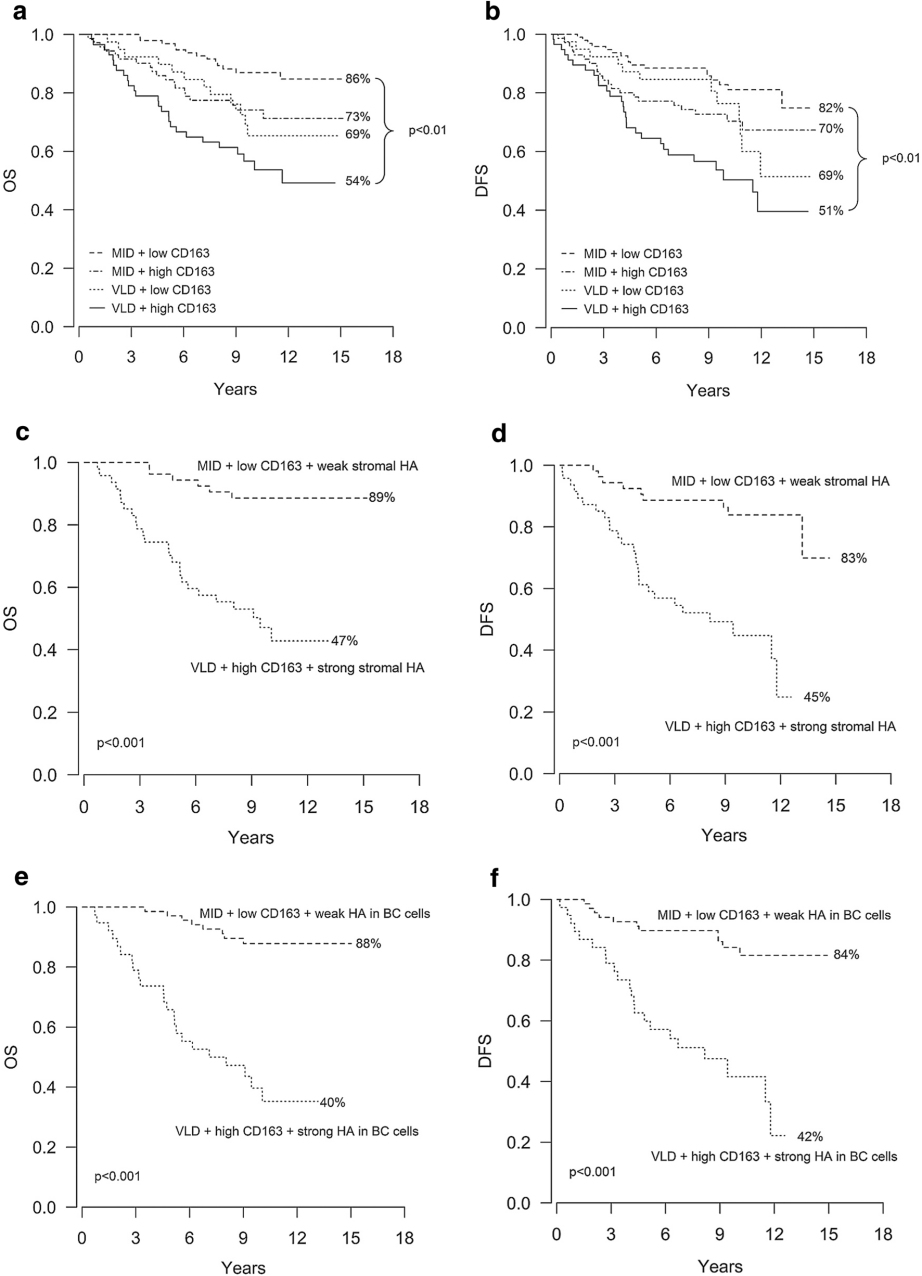
The combination of VLD breasts, abundance of M2-like TAMs and HA create a dismal survival. Kaplan–Meier curves showing overall survival (OS) and disease free survival (DFS) according to breast density and the level of M2-like (CD163+) TAMs (**a**, **b**); breast density, the level of M2-like (CD163+) TAMs and stromal HA (**c**, **d**) and breast density, the level of M2-like (CD163+) TAMs and HA in breast cancer cells (**e**, **f**)

In line with previous data [13, 26], OS and DFS were inferior in patients with VLD breasts as compared to MID breasts (*p* = 0.001 and *p* < 0.001), and with a high level of M2-like TAMs as compared to a low level (*p* = 0.001). Of the 57 patients with VLD breasts and a high level of M2-like TAMs, only 54% were alive at the end of the follow up as compared to 86% of the 95 patients with MID breasts and a low level of M2-like TAMs (*p* < 0.01); in other words, patients with VLD breasts and a high level of M2-like TAMs had a 4.4 times higher mortality risk (Fig. 1a, Table 4). Similarly, the risk for breast cancer recurrence was 3.7 times greater among patients with VLD breasts and a high level of M2-like TAMs as compared to patients with neither of these factors, and accordingly DFS rates were lower (Table 4, Fig. 1b). Patients with one of these unfavorable prognostic factors, i.e. VLD breasts or a high level of M2-like TAMs, had mediocre OS and DFS rates (Fig. 1a, b, Table 4).

**Table 4:** Survival analyses

	OS (%)	*p* value	HR	95% CI	DFS	*p* value	HR	95% CI
MID + low CD163	86				82			
MID + high CD163	73	0.026	2.23	1.10–4.51	70	0.053	1.88	0.99–3.57
VLD + low CD163	69	0.023	2.49	1.13–5.45	69	0.091	1.89	0.90–3.97
VLD + high CD163	54	< 0.01	4.39	2.25–8.54	51	< 0.01	3.71	2.03–6.79
MID + low CD163+ weak stromal HA	89				83			
VLD + high CD163+ strong stromal HA	47	< 0.001	6.48	2.65–15.82	45	< 0.001	5.52	2.47–12.32
MID + low CD163+ weak HA in BC cells	88				84			
VLD + high CD163+ strong HA in BC cells	40	< 0.001	7.79	3.47–17.48	42	< 0.001	5.66	2.71–11.82
BMI < 30	78				73			
BMI ≥ 30	56	0.001	2.32	1.42–3.81	62	0.044	1.67	1.01–2.77
T2D no	76				71			
T2D yes	57	0.027	1.90	1.07–3.37	66	0.480	1.25	0.67–2.31
BMI < 30 & no T2D	80				74			
Only T2D	59	0.056	2.19	0.98–4.91	59	0.2	1.68	0.76–3.71
Only BMI ≥ 30	57	0.001	2.58	1.44–4.62	57	0.011	2.08	1.18–3.67
BMI ≥ 30 & T2D	56	0.022	2.43	1.14–5.21	72	0.72	1.18	0.47–2.97

*OS* overall survival, *DFS* disease free survival, *MID* mixed density, *VLD* very low density, *HA* hyaluronan, *BC* breast cancer, *BMI* body mass index, *T2D* type 2 diabetes

Even poorer survival was seen among patients with VLD breasts, a high level of M2-like TAMs and high HA expression either in stromal (*n* = 47) or in breast carcinoma cells (*n* = 38), the risk for death being 6.5–7.8 times greater, and OS rates 40–47% versus 88–89%, when compared to patients with none of these factors (*p* < 0.001) (Fig. 1c, e, Table 4). The DFS rates were also dismal among patients with all three unfavorable factors (*p* < 0.001) (Fig. 1d, f, Table 4). Thus, HA abundance further increased the risk for an unfavorable outcome conferred by VLD breasts and a high level of M2-like TAMs. The differences in OS and DFS rates according to MBD, M2-like TAMs and tumor HA were similar among the HER2-positive (*n* = 129) and HER2-negative (*n* = 133) patients (Supplementary Table S2).

### Obesity and type 2 diabetes correlate with poor survival

**Fig. 2:**
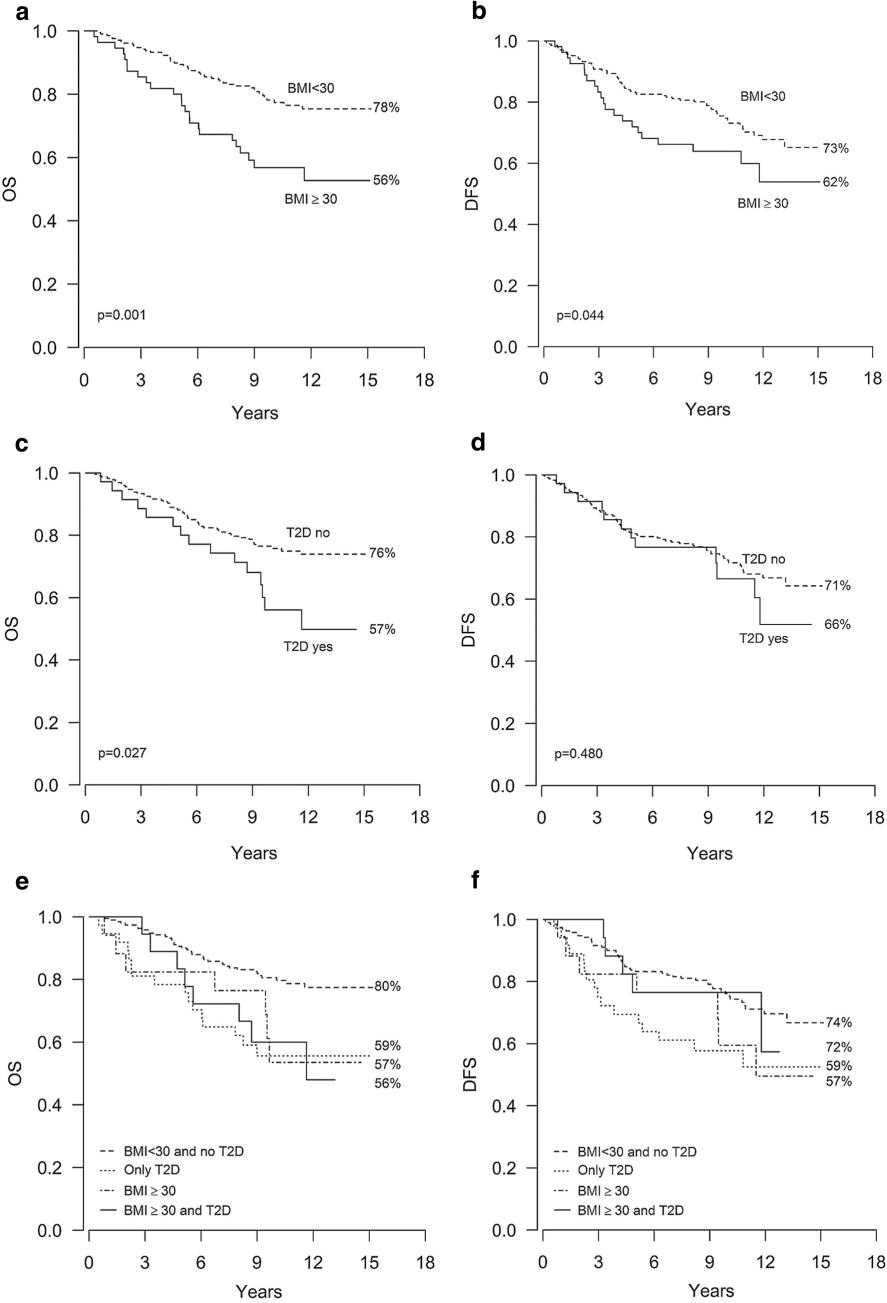
Obesity and type 2 diabetes correlate with poor overall survival. Kaplan–Meier curves showing overall survival (OS) and disease free survival (DFS) according to BMI (**a**, **b**), the presence/absence of T2D (**c**, **d**) and the presence/absence of obesity and T2D (**e**, **f**)

Both OS and DFS rates were inferior in the obese individuals as compared to the non-obese, 56% versus 78% for OS (*p* = 0.001) and 62% versus 73% for DFS (*p* = 0.044) (Fig. 2a, b, Table 4). BCSS was also inferior among the obese patients (*p* = 0.028, HR 1.94, 95% CI 1.07–3.49). The OS of the T2D patients was inferior as compared to the other subjects, as the OS rates were 57% versus 76%, respectively (*p* = 0.027) (Fig. 2c, Table 4). However, there were no statistically significant differences in the DFS rates (Fig. 2d, Table 4).

Of all the patients, 7% were both obese and had T2D, 14% were only obese, 6% had only T2D and 73% were non-obese and did not suffer from T2D. The OS rates were worse among patients who were only obese and among patients who were both obese and had T2D as compared to patients with neither of these conditions (*p* = 0.001 and *p* = 0.022, respectively), with a similar trend apparent in the patients with only T2D (*p* = 0.056) (Fig. 2e, Table 4). The DFS rate was inferior among the obese patients without T2D in comparison to patients with neither of these conditions (*p* = 0.011), but not among patients who had both obesity and T2D or only T2D (Fig. 2f, Table 4). In summary, while low OS correlated with both obesity and T2D, the DFS rate was inferior only among the obese patients without T2D.

### Cox multivariate analyses

In the Cox multivariate analysis, significant prognostic factors for OS were nodal status, VLD, tumor size, HER2 status, BMI and ER status. For BCSS, significant prognostic factors were nodal status, VLD, tumor size, HER2 status and M2-like TAMs (Table 5).

**Table 5:** COX multivariate analyses for OS and BCSS

	*p* value	HR	95% CI
OS			
Nodal status	< 0.001	3.78	1.99–7.21
VLD	< 0.001	2.93	1.72–4.97
Tumor size	< 0.001	2.6	1.53–4.42
HER2	0.022	1.83	1.09–3.08
BMI	0.032	1.86	1.06–3.26
ER	0.048	0.6	0.36–0.996
CD163+ TAMs	0.12	1.52	0.9–2.57
HA in BC cells	0.27	1.38	0.78–2.47
CD68 + TAMs	0.4	0.81	0.49–1.33
Stromal HA	0.43	1.34	0.65–2.77
T2D	0.71	1.13	0.59–2.18
BCSS			
Nodal status	< 0.001	7.8	2.97–20.43
VLD	< 0.001	3.25	1.77–5.95
Tumor size	0.001	2.99	1.54–5.81
HER2	0.022	2.06	1.11–3.83
CD163+ TAMs	0.03	2.02	1.07–3.83
ER	0.06	0.57	0.32–1.03
BMI	0.1	1.75	0.89–3.45
Stromal HA	0.22	1.81	0.71–4.64
HA in BC cells	0.42	1.32	0.68–2.55
T2D	0.66	0.83	0.36–1.93
CD68+ TAMs	0.67	0.88	0.49–1.58

*OS* overall survival, *BCSS* breast cancer specific survival, *VLD* very low density, *BMI* body mass index, *ER* estrogen receptor, *TAMs* tumor associated macrophages, *HA* hyaluronan, *BC* breast cancer, *T2D* type 2 diabetes

## Discussion

In this study of 262 breast cancer cases, we found a remarkable risk for death with the combination of fatty breasts (VLD), abundance of M2-like TAMs and tumor HA, demonstrating the importance of tumor microenvironment in breast cancer progression. We also showed that tumors in fatty breasts frequently contain high levels of M2-like macrophages, which facilitate pro-tumoral low-level inflammation. Fatty breasts associate with obesity and in the present study also with T2D, suggesting that the metabolic disturbance present in obesity and T2D may promote these local conditions favorable for tumor growth.

Tumors arising in fatty breasts are particularly abundant with HA [27] and here with M2-like TAMs, both indicators of chronic inflammation. M2-like macrophages facilitate low-level inflammation and tissue remodeling required for tumor growth [29]. HA in the peritumoral matrix promotes tumor cell invasion and shields them against immune attack, while HA fragments signal for an exacerbation of inflammation [22]. Moreover, HA synthesized under conditions of cellular stress can coalesce into cable-like structures that recruit macrophages [17] and induce their polarization towards the M2-phenotype [20]. What makes breast fat such a good platform for this cancer-promoting inflammation, remains a question to be answered in later studies.

Fatty breasts associate with obesity, and interestingly in the present study fatty breasts were found more often also in T2D patients even if they were not obese. Obesity associates also with a high level of M2-like TAMs [13] and tumor HA [23], and it is difficult to discern whether one of these factors is the primary effector in breast cancer progression with the other factors following as a consequence. In COX multivariate analyses including all of these factors, VLD displayed the highest significance. The key role of the peritumoral fat is further stressed by the fact that VLD remains as an independent indicator of survival even when adjusted for BMI, age and menopausal status [26]. Thus, obesity and T2D could be effectors that increase the fat content of the breasts and subsequently enhance pro-tumoral inflammation in the tumor microenvironment.

Nevertheless, there are many ways how obesity can promote breast cancer progression. Especially after menopause fat tissue is a major source of estrogen, a hormone known to promote breast cancer growth [30]. Also, higher leptin/adiponectin ratio may induce breast cancer growth [31], as well as hyperinsulinemia and elevated levels of insulin-like growth factor 1 (IGF-1) [32]. In addition, large tumor size, lymph node metastases, high tumor grade and possibly delayed diagnostics have been proposed to contribute to the unfavorable prognosis of obese patients [33]. However, in the present study, the standard clinicopathological parameters did not correlate with obesity, and obesity remained as an independent factor for poor OS also when adjusted for these known prognostic factors. Importantly, fat tissue content of HA, its CD44 receptor and accumulation of macrophages are characteristic features of the systemic inflammation that associates with obesity [9, 18]. Adipose tissue inflammation and insulin resistance associate with high CD44 expression [34], while weight loss reduces the expression of CD44 and the amount of macrophages in adipose tissue [35]. In addition, HA level in the blood is increased among obese [36] and T2D patients but, interestingly, not in type 1 diabetes [37]. This suggests that hyperglycemia alone does not account for the inflammation, thus leaving insulin resistance and hyperinsulinemia as possible culprits.

The metabolic dysfunctions such as insulin resistance and hyperinsulinemia that can subsequently develop into T2D are common in obesity. Indeed, in our study every third (33%, 18/55) obese patient had also T2D. Interestingly, the DFS rate was reduced among the obese patients, but not among the patients with both obesity and T2D. One explanation could be the administration of metformin, as T2D patients treated with metformin have a reduced incidence of several cancers, including breast cancer [38], and there is also evidence that metformin decreases breast cancer mortality [39, 40]. The higher DFS rate of obese patients with T2D compared to those only obese, suggest that metformin medication interferes with some key processes that promote breast cancer progression in obesity. Indeed, metformin not only decreases the levels of glucose and insulin in the circulation, but also reduces the uptake of glucose into cancer cells [41]. One characteristic of cancer cells is their very high glucose uptake and aerobic glycolysis (Warburg effect) [42]. The accumulation of glycolysis intermediates increases glucose flux into the hexosamine biosynthesis pathway resulting in increased level of its end product uridine diphosphate *N*-acetylglucosamine (UDP-GlcNAc) [14]. UDP-GlcNAc is a key substrate in HA synthesis and is involved in *O*-GlcNAcylation, the latter being a protein modification that contributes to cancer cell survival and associates with poor outcome in breast cancer [43]. There is evidence that metformin inhibits glucose consumption of breast cancer cells via reduced hexokinase activity [44] and decreases HA synthesis [45]. In addition, metformin can inhibit macrophage polarization into the M2-like phenotype in tumor microenvironment [46] and promote polarization into the M1-phenotype, resulting in the inhibition of tumor growth [47]. Recently it was shown in a rodent model, that metformin inhibited the progression of postmenopausal breast cancer and decreased the amount of macrophages in the tumor microenvironment [48]. Thus, the use of metformin may also explain why there were no correlations found between T2D and the amount of TAMs or HA in the present study. There was a non-significant trend towards lower tumor HA among T2D patients receiving metformin but the number of patients with metformin medication (*n* = 23) in the present study is too small to address this question properly. Prospective trials are ongoing in order to evaluate the effect of metformin on breast cancer outcome.

To conclude, in this study we showed that breast cancers in fatty breasts (VLD) often contain high levels of M2-like TAMs, suggesting that the readily available mammograms may provide important information of tumor biology and microenvironment. The dismal outcome among breast cancer patients with fatty breasts, a high level of M2-like TAMs and high tumor HA emphasize the importance of the local inflammatory conditions for tumor progression. Furthermore, the disturbed energy metabolism encountered in obesity and T2D may increase the fat-content of the breasts and subsequently promote local pro-tumoral inflammation, revealing a potential mechanism that predisposes these patients to a bleak prognosis.

## Electronic supplementary material

Below is the link to the electronic supplementary material.

Supplementary material 1 (DOCX 54 kb)

## Data Availability

The datasets generated and/or analyzed during the current study are not publicly available due to the fact that they contain information that could compromise research participant privacy but may be available from the corresponding author on reasonable request and with required permissions.
