# Supplementary material for: Tumor microenvironment and breast cancer survival: combined effects of breast fat, M2 macrophages and hyaluronan create a dismal prognosis
Source: Breast Cancer Res Treat. 2019 Nov 12;179(3):565–75. doi: 10.1007/s10549-019-05491-7 (PMC6997252; doi:10.1007/s10549-019-05491-7)
Supplement: Supplementary file 1 — Supplementary material 1 (DOCX 54 kb) [file 10549_2019_5491_MOESM1_ESM.docx]

**Supplementary Table S1** The correlations between TAMs, HA and T2D treatments

|  | **Metformin**  **n= 23**  **n (%)** | **No metformin**  **n= 11**  **n (%)** | **P value** |
| --- | --- | --- | --- |
| **CD163+ TAMs** |  |  |  |
| Low | 10 (44%) | 5 (46%) |  |
| High | 13 (56%) | 6 (54%) | 0.914 |
| **CD68+ TAMs** |  |  |  |
| Low | 13 (56%) | 6 (54%) |  |
| High | 10 (44%) | 5 (46%) | 0.914 |
| **HA in BC cells** |  |  |  |
| Weak | 13 (56%) | 4 (36%) |  |
| Strong | 10 (44%) | 7 (64%) | 0.271 |
| **Stromal HA** |  |  |  |
| Weak | 8 (35%) | 1 (9%) |  |
| Strong | 15 (65%) | 10 (91%) | 0.112 |

TAMs, tumor associated macrophages; HA, hyaluronan; T2D,

type 2 diabetes; BC, breast cancer

**Supplementary Table S2** Survival analyses stratified by HER2 status

| **HER2+ (n=129)** | **OS** | **P value** | **HR** | **95 % CI** | **DFS** | **P value** | **HR** | **95 % CI** |  |  |  |  |  |  |  |  |  |  |
| --- | --- | --- | --- | --- | --- | --- | --- | --- | --- | --- | --- | --- | --- | --- | --- | --- | --- | --- |
| **MID + low CD163** | 80 % |  |  |  | 84 % |  |  |  |  |  |  |  |  |  |  |  |  |  |
| **MID + high CD163** | 67 % | 0.15 | 2.12 | 0.76-5.97 | 67 % | 0.059 | 2.42 | 0.97-6.08 |  |  |  |  |  |  |  |  |  |  |
| **VLD + low CD163** | 63 % | 0.15 | 1.87 | 0.8-4.37 | 50 % | 0.013 | 3.64 | 1.32-10.05 |  |  |  |  |  |  |  |  |  |  |
| **VLD + high CD163** | 47 % | 0.002 | 3.67 | 1.62-8.32 | 47 % | 0.001 | 4.63 | 1.9-11.29 |  |  |  |  |  |  |  |  |  |  |
| **MID+low CD163+ weak stromal HA** | 82 % |  |  |  | 86 % |  |  |  |  |  |  |  |  |  |  |  |  |  |
| **VLD+high CD163+ strong stromal HA** | 41 % | 0.005 | 4.78 | 1.59-14.38 | 44 % | 0.005 | 5.86 | 1.69-20.38 |  |  |  |  |  |  |  |  |  |  |
| **MID+low CD163+ weak HA in BC cells** | 82 % |  |  |  | 85 % |  |  |  |  |  |  |  |  |  |  |  |  |  |
| **VLD+high CD163+ strong HA in BC cells** | 30 % | <0.001 | 6.46 | 2.3-18.1 | 40 % | 0.003 | 5.76 | 1.85-17.96 |  |  |  |  |  |  |  |  |  |  |
| **HER2- (n=133)** | **OS** | **P value** | **HR** | **95 % CI** | **DFS** | **P value** | **HR** | **95 % CI** |  |  |  |  |  |  |  |  |  |  |
| **MID + low CD163** | 92 % |  |  |  | 80 % |  |  |  |  |  |  |  |  |  |  |  |  |  |
| **MID + high CD163** | 81 % | 0.12 | 2.72 | 0.77-9.66 | 75 % | 0.438 | 1.45 | 0.57-3.66 |  |  |  |  |  |  |  |  |  |  |
| **VLD + low CD163** | 74 % | 0.43 | 3.7 | 1.04-13.14 | 83 % | 0.99 | 0.99 | 0.31-3.19 |  |  |  |  |  |  |  |  |  |  |
| **VLD + high CD163** | 63 % | 0.003 | 5.85 | 1.83-18.67 | 56 % | 0.012 | 2.92 | 1.26-6.79 |  |  |  |  |  |  |  |  |  |  |
| **MID+low CD163+ weak stromal HA** | 94 % |  |  |  | 81 % |  |  |  |  |  |  |  |  |  |  |  |  |  |
| **VLD+high CD163+ strong stromal HA** | 55 % | 0.006 | 8.55 | 1.85-39.64 | 45 % | 0.004 | 4.76 | 1.64-13.9 |  |  |  |  |  |  |  |  |  |  |
| **MID+low CD163+ weak HA in BC cells** | 93 % |  |  |  | 83 % |  |  |  |  |  |  |  |  |  |  |  |  |  |
| **VLD+high CD163+ strong HA in BC cells** | 50 % | 0.001 | 8.94 | 2.41-33.1 | 44 % | 0.001 | 5.14 | 1.92-13.76 |  |  |  |  |  |  |  |  |  |  |

OS, overall survival; DFS, disease free survival; MID, mixed density; VLD, very low density; HA, hyaluronan; BC, breast cancer
